# Neuropsychological attributes of urea cycle disorders: A systematic review of the literature

**DOI:** 10.1002/jimd.12146

**Published:** 2019-08-01

**Authors:** Susan E. Waisbren, Arianna K. Stefanatos, Teresa M. Y. Kok, Burcu Ozturk‐Hismi

**Affiliations:** ^1^ Division of Genetics and Genomics, Department of Pediatrics, Boston Children's Hospital Boston Massachusetts; ^2^ Department of Medicine, Harvard Medical School Boston Massachusetts; ^3^ Department of Child & Adolescent Psychiatry and Behavioral Sciences, Children's Hospital of Philadelphia Philadelphia Pennsylvania; ^4^ Horizon Therapeutics Plc Lake Forest Illinois; ^5^ Tepecik Education and Research Hospital Izmir Turkey

**Keywords:** intellectual disabilities, neuropsychological outcomes, urea cycle disorders

## Abstract

Urea cycle disorders (UCDs) are rare inherited metabolic conditions that impair the effectiveness of the urea cycle responsible for removing excess ammonia from the body. The estimated incidence of UCDs is 1:35 000 births, or approximately 113 new patients with UCD per year. This review summarizes neuropsychological outcomes among patients with the eight UCDs in reports published since 1980. Rates of intellectual disabilities published before (and including) 2000 and after 2000 were pooled and compared for each UCD. Since diagnoses for UCDs tended to occur earlier and better treatments became more readily available after the turn of the century, this assessment will characterize the extent that current management strategies have improved neuropsychological outcomes. The pooled sample included data on cognitive abilities of 1649 individuals reported in 58 citations. A total of 556 patients (34%) functioned in the range of intellectual disabilities. The decline in the proportion of intellectual disabilities in six disorders, ranged from 7% to 41%. Results from various studies differed and the cohorts varied with respect to age at symptom onset, age at diagnosis and treatment initiation, current age, severity of the metabolic deficiency, management strategies, and ethnic origins. The proportion of cases with intellectual disabilities ranged from 9% to 65% after 2000 in the seven UCDs associated with cognitive deficits. Positive outcomes from some studies suggest that it is possible to prevent or reverse the adverse impact of UCDs on neuropsychological functioning. It is time to “raise the bar” in terms of expectations for treatment effectiveness.

**Abbreviations**
ARGD: arginase deficiency
ASLD/ALD: argininosuccinate lyase deficiency
ASSD/ASD: argininosuccinate synthetase deficiency; citrullinemia type I
CI: confidence interval
CPS1: carbamoyl phosphate synthetase 1
CPS1D: carbamoyl phosphate synthetase 1 deficiency
CTLN2: citrullinemia type II
DQ: developmental quotient
FTTDCD: failure to thrive and dyslipidemia caused by citrin deficiency
HA: hyperammonemia
HHH: hyperornithinemia‐hyperammonemia‐homocitrullinuria;
ID: intellectual disability
IQ: intelligence quotient
NAG: *N*‐acetylglutamate
NAGSD: *N*‐acetylglutamate synthase deficiency
NBS: newborn screening
NCG: *N*‐carbamylglutamate
NICCD: neonatal intrahepatic cholestasis caused by citrin deficiency
OR: odds ratio
ORNT1: mitochondrial ornithine transporter
ORNT1D: mitochondrial ornithine transporter 1 deficiency
OTCD: ornithine transcarbamylase deficiency
RUSP: recommended Uniform Screening Panel
SD: standard deviation
UCD: urea cycle disorder
UCDC: Urea Cycle Disorders Consortium

## 1 INTRODUCTION

Urea cycle disorders (UCDs) are a set of rare inherited metabolic conditions that result in defects in urea cycle proteins responsible for removing excess ammonia from the body.^1^ The estimated overall incidence of UCDs in the United States and parts of Europe is 1:35 000 births, or approximately 113 new patients with UCD per year.^2^

**Figure 1:**
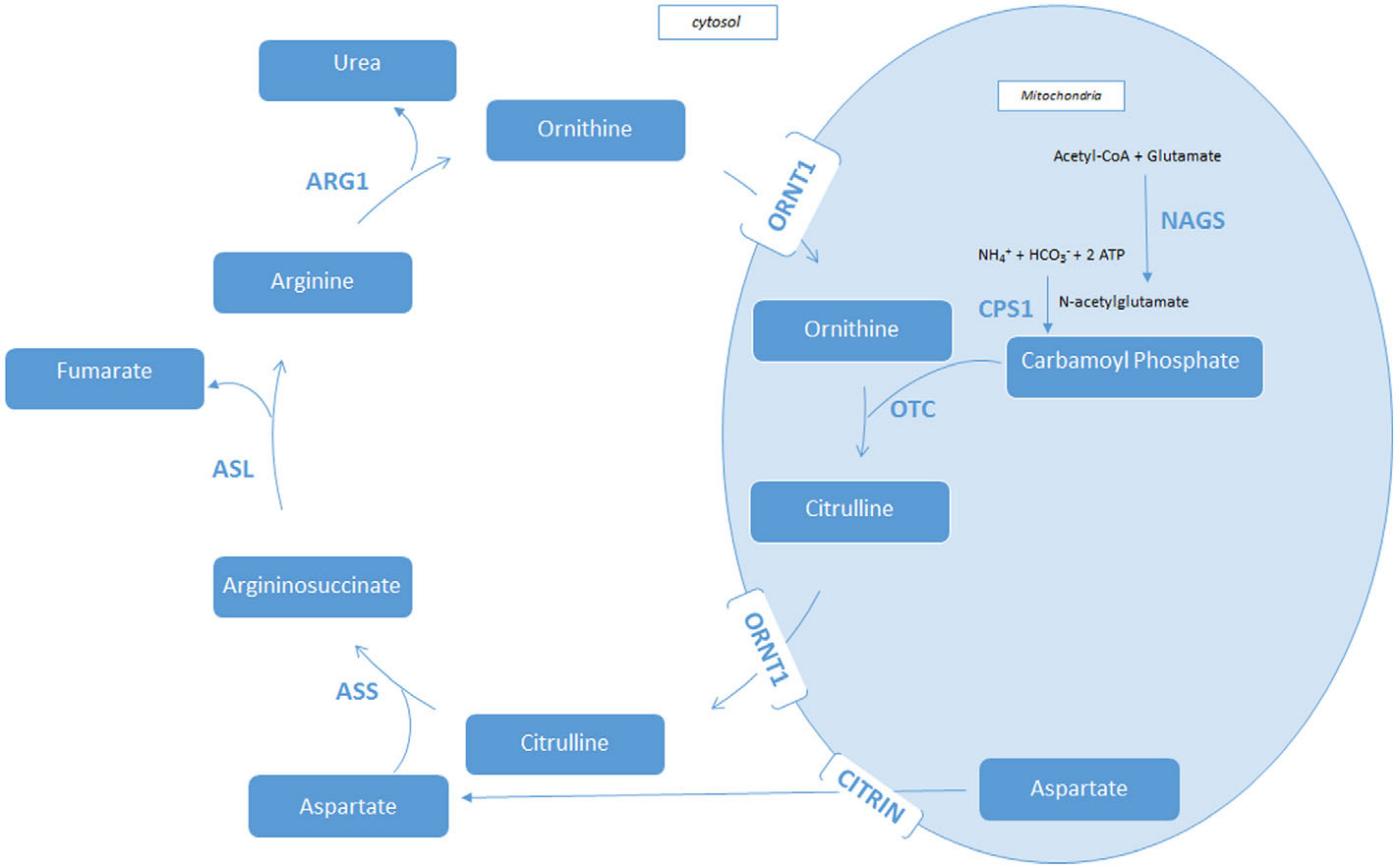
The urea cycle. Abbreviations: ARG1, arginase 1; ASL, argininosuccinate lyase; ASS, argininosuccinate synthetase; CPSI, carbamoyl phosphate synthetase 1; NAGS, *N*‐acetylglutamate synthase; OTC, ornithine transcarbamylase; ORNT1, mitochondrial ornithine transporter 1

Eight UCDs have been identified, each associated with defects in one of the six enzymes or two transporters of the urea cycle (Figure 1). The UCDs caused by enzyme deficiencies include proximal disorders, associated with altered or inhibited functioning of the mitochondrial enzymes: *N*‐acetylglutamate synthase (NAGS) deficiency (NAGSD; OMIM #237310), carbamoyl phosphate synthetase 1 (CPS1) deficiency (CPS1D; OMIM #237300), and ornithine transcarbamylase (OTC) deficiency (OTCD; OMIM #311250). The distal disorders, associated with altered functioning of the cytosolic enzymes, are argininosuccinate synthetase (ASS) deficiency /citrullinemia type I (ASSD; OMIM #215700), argininosuccinate lyase (ASL) deficiency (ASLD; ALD)/argininosuccinic aciduria (ASA; OMIM #207900), and arginase (ARG) deficiency/argininemia (ARGD; OMIM #207800). Defects in two mitochondrial transporters also lead to UCD: citrin deficiency (mitochondrial aspartate/glutamate carrier deficiency; OMIM #605814) and mitochondrial ornithine transporter 1 (ORNT1) deficiency (ORNT1D), also referred to as hyperornithinemia‐hyperammonemia‐homocitrullinuria (HHH) syndrome (OMIM #238970). Disruption of the urea cycle leads to insufficient detoxification of ammonia to urea and a subsequent buildup of ammonia in the brain and other tissues. Consequently, ammonia accumulation or increases in glutamine resulting from hyperammonemia (HA) lead to astrocyte swelling and cytotoxic brain edema.^3^

Apart from X‐linked OTCD, inheritance of all UCDs is autosomal recessive. Acute management of HA consists of renal replacement therapies to rapidly decrease blood ammonia levels, intravenous nitrogen scavenger drugs (sodium benzoate, alone or in combination with sodium phenylacetate, arginine),^4^ hydration, protein restriction, and treating catabolism with adequate calories from glucose, fats, and essential amino acids.^1^, ^5^ Long‐term management includes dietary treatment (protein restriction in combination with essential amino acids, vitamin, and mineral supplementation) and replacement of arginine and citrulline, if indicated. For UCDs other than NAGSD, ammonia/nitrogen scavenger drugs (sodium benzoate, sodium phenylbutyrate, and glycerol phenylbutyrate) are used to control ammonia levels.^5^, ^6^ *N*‐carbamylglutamate is a CPS1 activator that is used to replace NAGS in patients with NAGSD (and some with CPS1D). Liver transplantation is a reasonable long‐term management option for individuals with a UCD other than NAGSD.^5^, ^6^, ^7^, ^8^

This article presents a review of published studies with a focus on whether improvements in treatment have been successful in the prevention of intellectual disabilities in patients with UCDs. Since diagnoses tended to occur earlier and better treatments became more readily available after the turn of the century,^4^, ^9^ assessment results of cognitive functioning from studies published before and including the year 2000 (≤2000) and after the year 2000 (>2000) were compared for each UCD.

## 2 METHODS

The PubMed database was searched for articles reporting neuropsychological outcomes in patients with UCDs through July 1, 2018, using the search terms “urea cycle disorder” and the various names for the eight disorders. Bibliographies identified additional relevant articles. Only articles published after 1980 were included, since that was about the time ammonia scavenger drugs were first introduced.^10^ Variables considered were measures of cognitive functioning (eg, developmental quotients [DQ] and intelligence quotient [IQ]), age of symptom onset, age of diagnosis, treatment, and biomarkers. Age of symptom onset was defined as “early onset” (if HA or other symptoms occurred before 30 days of age) or “late onset” (if occurring at or after 30 days of age). When a case report or series of cases were updated, only the most recent publication was used. In total, 58 studies were used in the analyses presented in Table 1. Table S1a‐g presents data from studies used in the pooled results (some of which were not described in the text). The data are described in detail and respective studies, along with the additional references, are included in the Supplementary Table S1. Additional studies were referenced in the text, but excluded from the table if they provided information on behavioral or psychiatric issues. Pooled data for each disorder included the number of cases in the series, the number receiving a neuropsychological evaluation and/or description of cognitive functioning, and the number and percent with intellectual disabilities, defined as a DQ or IQ equal to or more than two SDs below the normative mean. Developmental tests and intelligence tests used in each study usually reported scores based on a normative mean of 100 with an SD of 15, as in the Wechsler IQ tests. Thus, intellectual disabilities were noted if the IQ/DQ was ≤70 on the Bayley Scales of Infant and Toddler Development or any of the Wechsler IQ tests. Less common were scores based on a normative mean of 50 ± 10 or z‐scores. The studies related to each disorder were pooled according to the year of publication unless the data collection time was recorded. For each disorder separately, odds ratios for intellectual deficiencies >2000 compared to ≤2000 were calculated along with 95% confidence intervals (CI) and *P*‐values using Fisher's exact test. We present descriptive statistics combining proximal disorders compared with distal disorders, but do not focus on these results, since outcomes vary among the different disorders.

**Table 1:** Summary of Cognitive Outcomes in Urea Cycle Disorders comparing published results before and including the year 2000 and after the year 2000

**Proximal disorders**								
Carbamoyl phosphate synthetase 1 (CPS1) deficiency								
	N _series_	N _CF_	N _ID_	% _ID_	N _neonatal_	N _late_	N _neonatal ID_	N _late ID_
≤2000	51	17	11	65%	14	3	9 (64%)	2 (67%)
>2000	76	52	21	40%	33	18	12 (36%)	10 (56%)
Odds ratio*** (95% CI) *P*‐value				0.38 (0.098, 1.311) *P* = 0.10			0.33 (0.068, 1.383) *P* = 0.11	0.64 (0.009, 14.443) *P* = 1
*N*‐acetylglutamate synthase (NAGS) deficiency								
	N _series_	N _CF_	N _ID_	% _ID_				
≤2000	10	10	5	50%				
>2000	37	22	2	9%				
Odds ratio*** (95% CI) *P*‐value				0.11 (0.008, 0.903) *P* = 0.02				
Ornithine transcarbamylase (OTC) deficiency								
	N _series_	N _CF_	N _ID_	% _ID_				
≤2000 Males	191	64	15	23%				
>2000 Males	153	145	32	22%	Excludes cases from Rüegger et al which did not specify how many males and females had ID			
Odds ratio*** (95% CI) *P*‐value				0.93 (0.440, 2.013) *P* = 0.86				
≤2000 Females	177	115	47	41%				
>2000 Females	404	403	59	15%	Excludes cases from Rüegger et al which did not specify how many males and females had ID			
Odds ratio*** (95% CI) *P*‐value				0.25 (0.152, 0.406) *P* < 0.001				
≤2000 All cases	368	179	62	35%				
>2000 All cases	675	666	128	19%	Includes cases from Rüegger et al which was excluded from the numbers reported for males and females separately above. Because of this, the sum of males and females reported for all cases (>2000) is less than the number reported here for all cases.			
Odds ratio*** (95% CI) *P*‐value				0.45 (0.308, 0.659) *P* < 0.001				
All proximal disorders								
	N _series_	N _CF_	N _ID_	% _ID_				
≤2000	429	206	78	37%				
>2000	788	740	151	20%				
Odds ratio*** (95% CI) *P*‐value				0.42 (0.298,0.597) *P* < 0.001				
**Distal disorders**								
Argininosuccinate synthetase (ASS) deficiency/citrullinemia type I								
	N _series_	N _CF_	N _ID_	% _ID_				
≤2000	88	50	34	68%				
>2000	156	137	51	37%				
Odds ratio*** (95% CI) *P*‐value				0.28 (0.131, 0.583) *P* < 0.001				
Argininosuccinate lyase deficiency (ASLD; ALD)/Argininosuccinic aciduria (ASA)								
	N _series_	N _CF_	N _ID_	% _ID_				
≤2000	67	42	23	55%				
>2000	232	212	131	62%				
Odds ratio*** (95% CI) *P*‐value				1.33 (0.644, 2.743) *P* = 0.39				
Arginase (ARG) deficiency / Argininemia								
	N _series_	N _CF_	N _ID_	% _ID_				
≤2000	8	4	4	100%				
>2000	28	24	15	63%				
Odds ratio*** (95% CI) *P*‐value				0 (0, 3.153) *P* = 0.27				
All distal disorders								
≤2000	163	96	61	64%				
>2000	416	373	197	53%				
Odds ratio*** (95% CI) *P*‐value				0.64 (0.392, 1.043) *P* = 0.07				
Mitochondrial transporter deficiencies								
	N _series_	N _CF_	N _ID_	% _ID_				
Citrin deficiency (mitochondrial aspartate/glutamate carrier deficiency)								
≤2000	0	0	0	‐‐	No studies reporting cognitive outcomes were found for Citrin deficiency ≤2000.			
>2000	138	137	4	3%				
Ornithine transporter 1 deficiency (ORNT1D); Hyperornithinemia‐hyperammonemia‐homocitrullinuria (HHH) syndrome								
≤2000	38	32	23	72%				
>2000	85	65	42	65%				
Odds ratio*** (95% CI) *P*‐value				0.72 (0.249, 1.947) *P* = 0.50				
**All disorders**								
	N _series_	N _CF_	N _ID_	% ID				
≤2000	630	334	162	49%				
>2000	1427	1315	394	30%				
Odds ratio*** (95% CI) *P*‐value				0.45 (0.353, 0.585) *P* < 0.001				

* N_series_, total number of cases reported in published studies included in this review; N _CF_, number of cases reporting on cognitive functioning; N _ID_, number of cases with intellectual disabilities; % _ID_, percentage of cases with intellectual disabilities; N _late_, number of late‐onset cases reporting cognitive functioning; N _late ID_, number of late‐onset cases with intellectual disabilities; N _neonatal_, number of neonatal‐onset cases reporting cognitive functioning; N _neonatal ID_, number of neonatal‐onset cases with intellectual disabilities; ≤ 2000, prior to and including the year 2000; > 2000, after the year 2000; CI, confidence interval.

^**^The estimated odds ratios (Fisher's exact test): Comparing rates of intellectual disabilities ≤2000 to >2000; 95% confidence intervals, and *P*‐values.

## 3 RESULTS

### 3.1 Proximal (mitochondrial) urea cycle disorders

#### 3.1.1 Carbamoyl phosphate synthetase 1 deficiency

CPS1D is rare, affecting approximately 1:150 000 to 1:200 000 individuals in the United States and Europe.^11^ CPSID is characterized by the early or late onset form. Neonates with CPS1D experience severe HA, and many die in the newborn period.^12^, ^13^ Survival rates may depend on age at onset of the first HA episode, availability of nitrogen‐reducing therapies, and occurrence of HA coma.^14^ Long‐term outcomes range from near‐normal functioning to severe psychiatric problems and cognitive deficits.^15^

For example, among two siblings with identical genotypes for CPS1D, one died as a neonate and the other received dietary therapy and functioned normally at age 45 years.^16^ Another child was rescued after fulminant neonatal‐onset but experienced severe global developmental delay.^17^ Three infants died despite treatment.^12^ In six infants with neonatal onset, one died and five exhibited developmental delay.^18^

Rüegger et al^19^ presented a series of 208 patients with “non‐classical” UCDs, defined as “…those with symptom onset after the newborn period; a mild disease course; uncommon presentations (at any age); asymptomatic individuals (screened because of affected relatives or identified through newborn screening, NBS) with biochemical features of a UCD”. The two individuals with CPS1D (one child and one adult) in this series had intellectual disabilities. In contrast, there was a case report of a baby diagnosed at 1 month of age with severe HA who received hemodialysis and exhibited “good” development at age 15 months.^20^ Kurokawa and colleagues reported on 18 patients who were identified because of clinical symptoms.^21^ Among the 15 neonatal‐onset cases, four survived but one who had severe intellectual disabilities died later and two did not have intellectual disabilities (one of whom had liver transplantation). Among the three late‐onset cases, one died at age 31 years at the time of diagnosis, one had intellectual disabilities (diagnosed at age 45 years), and one diagnosed at age 13 years performed above that range.

In another series, 12 children with neonatal‐onset and 3 adults with late‐onset CPS1D were identified.^22^ Among the early‐onset cases, nine died before the age of 2 years and 1 one died at the age of 4 years. Two children developed normally: one aged 3 years with liver transplantation and one aged 2 months awaiting transplantation. Two of the adults had intellectual disabilities and one had intermittent HA.

Outcomes varied among the 22 cases with CPS1D enrolled in the UCDC Longitudinal Study.^23^ The mean Bayley Cognitive Composite score (similar to a DQ) among the 10 infants receiving evaluations was 77 (SD = 17), ranging from 60 to 85. Four of the five children evaluated at preschool age demonstrated behaviors common in children with autism spectrum, but none of the nine preschool‐ or school‐aged children evaluated had an IQ <71. Two adults with CPS1D performed in the range of intellectual disability (IQ of 66 and 62) and two others performed in the average range (IQ of 105 and 86).

Mild deficiencies lead to later or even adult‐onset CPS1D. In one study, two children with late‐onset CPS1D first exhibited signs of psychiatric disturbances, “delirium” in one case and autistic behaviors in the other, which improved with dietary treatment, although one had intellectual disabilities.^24^ A young woman with mild learning disabilities experienced intermittent nausea, vomiting, and psychosis (excitation, aggression, and insomnia) during her menses since age 13.^25^ At age 18, she presented with coma and CPS1D was finally diagnosed. She recovered from the coma but performed in the range of intellectual disabilities thereafter.

Another woman with CPS1D, who had a history of learning disabilities and several episodes of disorientation, was not diagnosed until experiencing a severe HA episode with coma during pregnancy.^26^ Treatment led to symptom resolution and baseline functioning, although no neuropsychological testing was performed. The baby was born at 22 weeks and died.

Foschi et al^7^ described the case of a 17‐year‐old girl who was diagnosed with CPS1D at age 3 years. Before liver transplantation, she had intermittent attacks characterized by somnolence, ataxia, coordination defects, and temporal cognition distortion. Following liver transplantation, she led a normal life, with good clinical condition and no dietary restrictions or hospitalizations.

Another case report described a 49‐year‐old man with late‐onset CPS1D who experienced a stroke and HA encephalopathy.^27^ The HA episodes resolved with liver transplantation, and the patient was able to return to work.

According to studies published ≤2000 with data on cognitive functioning, 65% of surviving patients with CPS1D had intellectual disability, and the prevalence of intellectual disabilities was 64% and 67% among neonatal‐onset and later‐onset individuals, respectively. The rate of intellectual disability for children would have been much higher, yet at least one‐third of early‐onset children died in infancy or shortly thereafter with neurological symptoms. The prevalence of intellectual disabilities declined after 2000 in both neonatal‐ and late‐onset patients (36% and 56%, respectively).

#### 3.1.2 *N*‐acetylglutamate synthase deficiency (NAGSD)

A defect in the next step of the urea cycle leads to NAGSD—the rarest of the UCDs—with an estimated incidence of less than 1:2 000 000 in the United States and Europe.^2^ NAG is synthesized by the mitochondrial enzyme NAGS and activates CPS1. Without NAGS, there is no activity of CPS1 and, hence, clinical and biochemical features of NAGSD are indistinguishable from CPS1D, with increased plasma ammonia and glutamine, decreased plasma citrulline, and normal to low levels of urinary orotic acid. Adult patients are rare and seldom recognized. Symptoms include a self‐selected low‐protein diet, headaches, vomiting, lethargy, behavioral changes, confusion, altered consciousness, combativeness, and encephalopathy.^28^ Treatment with *N*‐carbamylglutamate has been recommended as the treatment of choice for NAGSD, allowing for an unrestricted diet and no other medications except during a metabolic crisis.^29^

Bachmann et al reported on the first two cases of NAGSD, both of whom died in infancy with severe neuropathy associated with HA.^30^ Another case report described early‐onset symptoms and severe intellectual disability and motor delay; the child died at the age of 9 years.^31^ Pandya et al reported on two brothers, one of whom appeared normal at 18 months and the other who exhibited developmental delays at 6 months.^32^ The next published case presented at age 2 months and experienced severe intellectual disability and cortical blindness by age 2 years despite treatment.^33^ Other early cases represented a mixed set of outcomes.^32^, ^34^, ^35^, ^36^, ^37^, ^38^, ^39^

Later reports suggested a positive impact with prompt diagnoses via genetic testing and treatment initiation.^40^, ^41^, ^42^

Sancho‐Vaello et al^43^ reviewed genetic mutations in published and unpublished cases (56 patients from 42 families). In this series, there were 17 previously unpublished cases with outcome data. Among the 10 patients with neonatal‐onset disease, six died within 20 days, three had a normal outcome, and one had developmental delay even with treatment. Of the seven late‐onset cases, one died without treatment and six had a normal outcome.

All four participants with NAGSD (one child and three adults) in the UCDC Longitudinal Study performed within the average range of intellectual functioning (IQ of 87‐116).^23^ One woman who was undiagnosed until adulthood experienced serious psychiatric problems and cognitive impairment before her diagnosis and treatment for NAGSD.

Pooled data of the 10 cases reported ≤2000 revealed intellectual disabilities in five (50%). Two of the 22 cases (9%) described after 2000 experienced intellectual disabilities, although neonatal death continued to be a common occurrence.

#### 3.1.3 Ornithine transcarbamylase deficiency

OTCD is the most common type of UCD, affecting approximately 1:56500 individuals.^2^ Clinical severity is variable. As inheritance of OTCD is X‐linked, the degree of enzyme deficiency and neuropsychological impairment often differs between males and females, with females being less affected or asymptomatic. Infant boys often present with life‐threatening HA within the first hours of life, and nearly half do not survive.^44^ Thus, studies reporting on neuropsychological outcomes in OTCD are inherently biased by the exclusion of the most severely affected male patients.

Msall and colleagues reported that two of five (40%) male patients with OTCD had intellectual disabilities (IQ <70) with a mean IQ of 56 (SD = 18).^45^ An additional two male patients with severe neurological involvement died before age 1 year. Among heterozygous females with OTCD, intellectual disabilities were found in five of 13 (38%) in one study^46^ and in five of 10 (50%) in another study (mean IQ = 61; SD = 66).^47^

Uchino et al^48^ reported outcome data for 140 patients with OTCD. The 5‐year survival rate was 30% for males and 39% for females. Among patients who survived 5 years after symptom onset, 3 of 27 (11%) males and 5 of 19 (26%) females had intellectual disabilities. All patients whose highest recorded ammonia concentration was <180 μmol/L were neurologically intact, whereas all patients whose peak ammonia concentration was >350 μmol/L sustained severe brain damage or died.

Nassogne et al^49^ summarized outcomes in 150 individuals identified with OTCD from 1972 to 2000. All but one of the 66 boys with neonatal‐onset OTCD died. Chronic psychiatric problems were the predominant symptom before diagnosis in 4 of the 46 late‐onset boys and 2 of the 36 late‐onset girls. Agitation, confusion, aggression, and irritability were often noted. Of the surviving boys, nearly one‐half had “moderate” difficulties, whereas 20% of the surviving girls were described as being severely affected.

Another series included long‐term outcomes of 28 surviving children (boys, n = 5; girls, n = 23) assessed at one clinic.^50^ This sample included 4 neonatal‐onset (boys, n = 1; girls, n = 3) and 20 late‐onset cases (boys, n = 3; girls, n = 17). Overall, 50% of individuals performed in the range of intellectual disabilities (boys, n = 1; girls, n = 13).

In the Bachmann et al^51^ sample that included 36 individuals with OTCD, all those with an initial ammonia level > 300 μmol/L or a maximum ammonia level > 480 μmol/L had intellectual disabilities.^51^ Krivitzky et al^52^ reported intellectual disabilities in 20% of individuals with OTCD participating in the UCDC Longitudinal Study. An updated report from the UCDC presented data on different age cohorts.^23^ Infant girls (n = 25) had a mean Bayley Cognitive Composite score of 96 (SD = 16) and 13% had a DQ <71. In contrast, infant boys (n = 27) had a mean DQ of 74 (SD = 19) and 52% had a DQ <71. The preschool cohort of 31 girls with OTCD performed well, with a mean Full Scale IQ of 105 (SD = 15), and none performed in the range of intellectual disabilities. The 15 preschool age boys in this cohort attained a mean Full Scale IQ of 91 (SD = 22) and 13% had an IQ <71. By school age (6‐16 years), 8% of the 97 girls and 9% of the 34 boys with OTCD had an IQ <71. The adult OTCD cohort comprised 156 women and 25 men. The women attained a mean Full Scale IQ of 102 (SD = 16) and 5% had an IQ <71, while the men attained a mean Full Scale IQ of 101 (SD = 21) and 15% had an IQ <71.

In the Rüegger et al^19^ study of individuals with “non‐classical” UCDs, intellectual disabilities were identified in 37 of 121 individuals (31%) with OTCD. Overall, 64% of the 83 females and 71% of the 38 males in the sample had experienced ≥1 HA episode, a far greater percentage than those with intellectual disabilities, suggesting that HA alone does not necessarily lead to severe long‐term cognitive effects.

Martín‐Hernández et al^53^ summarized registry data from 26 males and 41 females. Although neuropsychological assessments were not completed for most patients, 45% exhibited signs of neurologic damage (males, n = 7; females, n = 22). Other studies showed poor executive functioning, working memory, fine motor abilities, and academic achievement.^23^, ^54^

Several studies suggest that intellectual abilities may not be affected if ammonia levels do not reach toxic levels (generally defined as >100 μmol/L). In a study of 19 mildly symptomatic or asymptomatic women, all participants had intellectual abilities within the average range and performed as well as or better than controls in most domains.^55^ However, they showed relative weaknesses in fine motor skills and nonverbal domains. Another study that included primarily asymptomatic individuals without a history of HA episodes revealed no significant difference in IQ between patients with OTCD and a group of individuals without OTCD.^56^ However, subtle deficits in fine motor coordination, executive functioning (especially working memory), and cognitive flexibility were found, albeit only when the cognitive load was relatively high. Self‐reported difficulties in social relationships, as well as anxiety and depression, have also been described in asymptomatic adults with OTCD.^23^

Although mortality rates may not have greatly improved, some studies reported improvements in cognitive outcomes as treatment paradigms have encompassed ammonia scavenging drugs as well as dietary treatment and early intervention. The pooled rate of intellectual disabilities among females declined from 41% in studies published ≤2000 to 15% >2000. Among males, pooled rates of intellectual disabilities in studies published ≤2000 and > 2000 were 23% and 22%, respectively.

Overall, 24% of the individuals with proximal UCDs had intellectual disability, and the rate of intellectual disability declined (37% ≤2000 vs 20% >2000).

### 3.2 Distal (cytosolic) urea cycle disorders

#### 3.2.1 Argininosuccinate synthetase deficiency/citrullinemia type I

Estimated rates of ASSD/citrullinemia type I range from 1:57 000^57^ to 1:250 000 newborns.^2^ ASSD is characterized by elevated citrulline (usually 1000 μmol/L, where normal is <50 μmol/L) and absence of argininosuccinic acid. Ammonia is elevated above 1000 μmol/L in the neonate, where normal is 40‐100 μmol/L. Arginine tends to be in the low to normal range. Glutamine, a surrogate marker for ammonia, tends to be increased as well.^58^ In ASSD, serum glutamine, as compared with serum ammonia, appears to be more closely related to the level of the diffuse pattern of cerebral involvement on magnetic resonance imaging.^59^ Both the severe neonatal or “classic” form and late‐onset form present with HA, although symptoms may be subtle in the later onset form. Symptoms include lethargy, headache, slurred speech, and somnolence. Liver failure also occurs in some patients.^60^ Without treatment, symptoms can progress to seizures, coma, and death. ASSD is now included in the Recommended Uniform Screening Panel that has been adopted in the United States. With early identification, treatment with a protein‐restricted diet and nitrogen scavenger therapy (if needed) can often be initiated before symptom onset. Milder cases detected by NBS may be asymptomatic throughout life or present much later with HA.^19^ Liver transplantation corrects the underlying disorder but does not increase arginine levels or reverse neurologic dysfunction.^58^

In the Nassogne series focusing on 33 patients identified from 1972 to 2000, 14 individuals survived.^49^ One of the seven early‐onset cases and two of the seven late‐onset cases had severe neurological symptoms.

An 8‐year‐old boy who presented with HA coma at 8 days of age was treated with protein restriction, arginine, and sodium benzoate. He achieved average cognitive abilities and high‐average scores on gross motor skills, personal‐social, and performance domains of the Griffiths Mental Developmental Stage assessment.^61^ His language score was average, although some vocabulary and fluency difficulties and fine motor weaknesses were noted.

Three patients diagnosed with ASSD through NBS demonstrated a normal neurodevelopmental profile.^62^ Karall et al described the case of a 14‐year‐old boy with late‐onset ASSD who presented with seizure and confusion that resolved upon treatment for elevated ammonia levels.^63^ Later, he had normal neurocognitive development and received no ongoing treatment for ASSD. Another case report described moderate to severe intellectual disabilities in a 31‐year‐old woman who presented with severe HA at neonatal onset and had subsequent episodes of HA and seizures, despite treatment with protein restriction, arginine, and ammonia scavenger drugs.^64^

In an analysis of patient data from the UCDC Longitudinal Study, rates of intellectual disabilities were: 18% for the 44 infants and toddlers (ages 6 months to 3 years); 18% among the 23 preschool children age (4‐5 years old); 17% among 42 school‐age children (6‐16 years); and 26% among 19 adults.^23^ Delays or deficits were noted most commonly in the motor and memory domains. Two preschool children were rated at risk of autism spectrum disorder. Attention deficits were common in the school‐age children. The follow‐up study of enrollees aged ≥3 years identified 35% of 64 patients with intellectual disabilities.^65^

Lee et al^66^ compared neuropsychological outcomes in 14 patients with ASSD in terms of age at symptom onset. Among patients with neonatal‐onset ASSD (n = 12), 3 died, four had severe intellectual disabilities, four had moderate intellectual disabilities, and one developed normally. The three patients with late‐onset symptoms had three different outcomes: one died, one had mild intellectual disabilities, and one developed normally. Patients identified presymptomatically (n = 3) fared the best, as all three had normal development and intellectual abilities within the average range.

Across six pooled studies published ≤2000, 34 of 50 patients (68%) with ASSD had intellectual disabilities, whereas the rate declined to 37% (n = 51/137) in eight studies published after 2000.

#### 3.2.2 Argininosuccinate lyase deficiency/Argininosuccinic aciduria

ASLD/ASA is the most common distal disorder. ASLD affects approximately 1:49 000^67^ to 1:70 000 newborns.^68^ It is characterized by elevated citrulline and argininosuccinic acid and can lead to HA. Patients without a history of HA may experience seizures and developmental delay.^67^ Other risks include hepatic disease and systemic hypertension.^69^, ^70^ Among UCDs, ASLD is the least likely to result in neonatal death.^71^ Treatment involves a protein‐restricted diet and supplemental arginine. NBS for ASLD initially took place for a limited time in Massachusetts, U.S., from 1969 to 1978^67^ and in Austria from 1978 to 2001.^68^ ASLD later became part of the RUSP (NBS, 2006), leading to the identification of many more cases with mild forms of the condition.^72^ Liver transplantation corrects the deficiency and may prevent neurologic declines, as well.^73^

Rates of intellectual disabilities ranged from 83% in a 1998 study^48^ to 6% in a study of individuals identified by NBS.^68^ Among 20 patients in one series, 15 survived long enough to be evaluated, and 9 of these 15 were noted to have moderate to severe intellectual disabilities.^74^ Frequency and severity of HA episodes were not related to outcomes. Tuchman et al^75^ reported that patients with ASLD enrolled in the UCDC study experienced intellectual disability (70%), learning disability (56%), attention‐deficit/hyperactivity disorder (30%), communication disorder (14%), and seizures (32%). In a recent analysis of cross‐sectional data from the UCDC study, particular deficits were noted in fine motor skills, attention, memory, and social interactions.^65^ The percentage of study participants who performed in the range of intellectual disabilities increased with each age cohort: 23% of infants, 24% of preschool children, 38% of school‐age students, and 52% of adults. The mean Full Scale IQ among the 65 individuals aged ≥3 years in the UCDC study was 72 (SD = 21).

Outcomes among individuals identified by NBS may be better, although still somewhat variable. After 13 to 33 years of follow‐up, 8 of 13 individuals identified by NBS had normal intellectual and psychomotor development, while 5 had learning disabilities and borderline IQ (≤85).^67^ Although all patients were initially treated with protein restriction, four of five patients with learning disabilities had discontinued the low‐protein diet early. A similar longitudinal study followed 23 patients diagnosed with ASLD through NBS.^68^ IQ data were available for 17 patients (mean age = 12.4 years); 11 patients had IQs of 90‐136, 5 had IQs of 80‐89, and 1 patient had intellectual disabilities, with an IQ of 61. A case report described a 26‐month‐old male with ASLD who was missed by NBS and presented with nonresponsive focal seizures and experienced intellectual disability, hyperactivity, and severe expressive language delay.^76^

A retrospective analysis compared neuropsychological outcomes between individuals with neonatal‐onset (n = 8) and late‐onset (n = 3) ASLD.^77^ Among neonatal‐onset individuals, the DQ/IQ score was near normal at diagnosis but declined slowly thereafter, stabilizing after the age of 4‐6 years. In contrast, individuals with late‐onset ASLD presented with mild to moderate intellectual disabilities at diagnosis with little change over the years. All late‐onset patients had a seizure disorder, whereas three of eight neonatal‐onset individuals had seizures, suggesting a potentially beneficial effect of earlier onset (or earlier recognition of symptoms) and treatment. In contrast, a study of 56 individuals with variable onset of ASLD (early onset, n = 23; late onset, n = 23; newborn screened, n = 10) reported similar long‐term outcomes in all three groups, with a similar neurological phenotype regardless of symptom onset.^78^ In this study, 48 of 52 study participants had mild or moderate intellectual disabilities, primarily affecting speech and learning.

Across seven pooled studies published ≤2000, 23 of 42 (55%) patients had intellectual disabilities and the prevalence of intellectual disabilities increased to 62% (n = 131/212) in 10 studies >2000.

#### 3.2.3 Arginase deficiency (ARGD)/Argininemia

ARGD interferes with the breakdown of arginine to urea and ornithine and represents the last step in the urea cycle. The incidence is estimated at 1:950 000 newborns in the United States and Europe.^2^ ARGD is characterized by elevated plasma arginine (2‐5 × normal) and is not usually associated with HA in the neonatal period, although HA may occur at any age.^79^ Spastic diplegia is the most common presenting symptom.^80^ When treated early, ARGD had not been thought to be associated with early developmental delays.^81^ However, more recent reviews indicate that cognitive and motor declines persist and hepatomegaly develops in some children.^82^ Liver transplantation for ARGD corrected arginine abnormalities in one case.^83^

The first reported case with neuropsychological testing had an IQ of 70.^47^ A retrospective study examining neuropsychological outcomes in patients with UCDs included seven patients with ARGD. The three patients who survived 5 years had severe intellectual disability (IQ <34).^48^

One case report described a 4‐year‐old girl who presented with autistic‐like symptoms and was late diagnosed with ARGD. Her autistic behaviors and hyperactivity resolved after 1 year of treatment with arginine and sodium benzoate plus a protein‐restricted diet, although her IQ was 72.^84^ A boy with progressive gait abnormalities and pyramidal signs was diagnosed with ARGD at 10 years of age.^85^ Despite 7 months of treatment with a protein‐restricted diet and sodium benzoate, his IQ declined from 94 to 82.

Among participants in the UCDC Longitudinal Study, children aged ≤3 years (n = 3) had a mean DQ of 88, and none were noted to have intellectual disabilities.^23^ One of the preschool children (age 4‐5 years) scored high on an autism spectrum disorder scale, and 33% of the school‐age children (ages 6‐16 years, n = 6) performed in the range of intellectual disabilities. In a more recent analysis of patient data from the UCDC study, 67% (n = 8/12) of those aged ≥3 years had intellectual disabilities, with a mean IQ of 65 (SD = 12).^65^

Martín‐Hernández and colleagues^53^ included two cases of ARGD in their NBS follow‐up study. Neither child performed in the range of intellectual disabilities. In contrast, Rüegger et al reported that seven of eight children identified by NBS experienced intellectual disabilities.^19^

Pooled data indicate that 100% (n = 4/4) of children included in reports on ARGD ≤2000 performed in the range of intellectual disabilities, and 63% (n = 15/24) of individuals attained IQ within this range after that time.

Overall, the rate of intellectual disability was 55% among distal UCDs. The rate of intellectual disability derived from studies ≤2000 was 64% vs 53% >2000.

### 3.3 Urea cycle mitochondrial transporter defects

#### 3.3.1 Citrin deficiency

Citrin is a mitochondrial aspartate/glutamate carrier providing aspartate for the first cytosolic reaction of the urea cycle. Citrin deficiency generally presents as one of two major clinical pictures: neonatal intrahepatic cholestasis caused by citrin deficiency (NICCD) or adult‐onset citrullinemia type II (CTLN2). Failure to thrive and dyslipidemia caused by citrin deficiency (FTTDCD) is a third intermediate form of the disease seen in older children.^86^ The neonatal form is unique among the UCDs in that early symptoms abate or completely disappear within the first year of life, and later symptoms do not arise until adulthood. Generally thought to be extremely rare outside of East Asia (mostly Japan),^87^ this UCD has reported incidence rates of 1:100 000 to 1:230 000.^86^ However, a study in Texas in which 10 patients received genetic testing found evidence for Arabic, Pakistani, French Canadian, and Northern European family origin.^88^ NBS with tandem mass spectrometry now takes place in Japan and Taiwan.^89^, ^90^

In the neonatal period, the buildup of bile caused by NICCD is associated with failure to thrive and dyslipidemia.^86^ Early signs may involve a “chubby face,” which resolves after 1 year.^91^ Other clinical symptoms may include hypoglycemia, pancreatitis, fatigue, and anorexia.^86^ In NICCD, almost all signs of failure to thrive and liver disease disappear within the first year of life after treatment with medium‐chain triglyceride‐rich formula or lactose‐free formula (for those with secondary galactosemia). In a follow‐up study of 51 children with NICCD,^92^ evidence of liver abnormalities and failure to thrive were noted, but only one child had intellectual disabilities. Hutchin et al^87^ reported on two children with early liver disease but no cognitive or other clinical manifestations after 1 year of age.

The UCDC study included two children with NICCD who received neuropsychological evaluations.^23^ During infancy, the children experienced gross motor delay but normal cognition. During preschool, both performed well within the average range, but verbal skills were relatively poorer than nonverbal abilities. In one child evaluated at school age (9 years old), there were no indications of cognitive or behavioral consequences of this condition In a screening of children with neurodevelopmental disabilities, one child with NICCD was identified at age 16 months and subsequently died at 22 months of age because of liver failure and one child had transient developmental delays but improved with age.^93^

Citrin deficiency becomes truly problematic in adulthood as CLTN2, which is characterized by high plasma citrulline and ammonia levels with recurrent encephalopathy. Patients with CLTN2 typically present with HA and some experience recurrent neuropsychiatric episodes, including behavior disturbances, nocturnal delirium, disorientation, altered consciousness, convulsive seizures, and coma.^86^ It is critical to distinguish CTLN2 in a patient with HA coma, as its treatment is unique. Unlike treatment for other UCDs, introduction of a high amount of glucose and low amount of protein can be fatal. Asking for the patient's dietary history can be lifesaving, as carbohydrate avoidance and fat‐craving are typical eating habits seen in individuals with CTLN2. Treatment for CTLN2 consists of a diet high in fat and protein, low in carbohydrates, supplemented with arginine, medium‐chain triglycerides, and sodium pyruvate.^94^ Liver transplantation had been the choice of treatment for CTLN2 to eliminate recurrent HA, but oral sodium pyruvate supplementation is now deemed safer and equally effective when started early.^95^, ^96^

One case report described an adult who was diagnosed with schizophrenia for many years before finally receiving the correct diagnosis after her HA was recognized. Once treated for her metabolic disorder, her symptoms resolved.^97^

Other case reports describe HA and a variety of associated symptoms, including headache, confusion, aggression, delusions, seizures, and coma in the postpartum period or following anesthesia or gastrectomy.^98^, ^99^, ^100^

Although cases of NICCD and CTLN2 were described before 2000,^101^, ^102^ no data on cognitive functioning were reported. Pooled data on studies published after 2000 are presented in Table 1G. A small number of NICCD cases (n = 3/126, 3%) were noted to have intellectual disabilities.

#### 3.3.2 Mitochondrial ornithine transporter 1 deficiency/Hyperornithinemia‐hyperammonemia‐homocitrullinuria syndrome

HHH syndrome is caused by impaired ornithine transport across the inner mitochondrial membrane due to mutations in the ORNT1 transporter.^103^ HHH syndrome is associated with high clinical variability; mild forms manifest with learning difficulties and slight neurological involvement, while symptoms of severe deficiencies include coma, lethargy, hepatic signs, and seizures. Long‐term management consists of a low‐protein diet, citrulline, or arginine supplementation, and, in some patients, nitrogen‐scavenging drugs. Although the incidence of HHH syndrome is unknown, it is estimated at <1:2 000 000.^2^

Martinelli et al^103^ published a comprehensive review of previously published cases as well as new cases of HHH syndrome and examined neuropsychological outcomes in 111 patients. The neuropsychological profile of patients was characterized by abnormal behavior (n = 30/51), intellectual disability (n = 58/85), seizures (n = 31/88), lethargy (n = 47/79), and coma (n = 30/94). Among patients with intellectual disability, 36% had mild or moderate intellectual disability (IQ/DQ of 35‐69), while 38% had severe disability (IQ/DQ <35). This series included data from Kim et al^104^ who reported on longitudinal follow‐up of four adults with HHH syndrome. Normal development was reported during infancy and early childhood in all four cases; however, by adulthood, all experienced progression of neurological symptoms and intellectual disability despite attempts at treatment.^104^

In the UCDC Longitudinal Study,^23^ three of four infants demonstrated developmental delay but performed within the borderline to average range of intelligence by preschool and school age. The one adult attained an IQ of 100 at age 21 and 84 at age 26 years. Guan et al^105^ reported on three cases of HHH syndrome identified because of clinical symptoms from 2011‐2016. All three experienced developmental delay, and two had moderate intellectual disabilities. Once treated, improvements were noted in behavior and functioning

Pooled data, primarily gleaned from the Martinelli et al^103^ review, suggest modest improvements when treatment is initiated early, but the disease may progress in adulthood. Intellectual disabilities were reported in 72% and 65% of published cases ≤2000 and > 2000, respectively.

## 4 DISCUSSION

**Figure 2:**
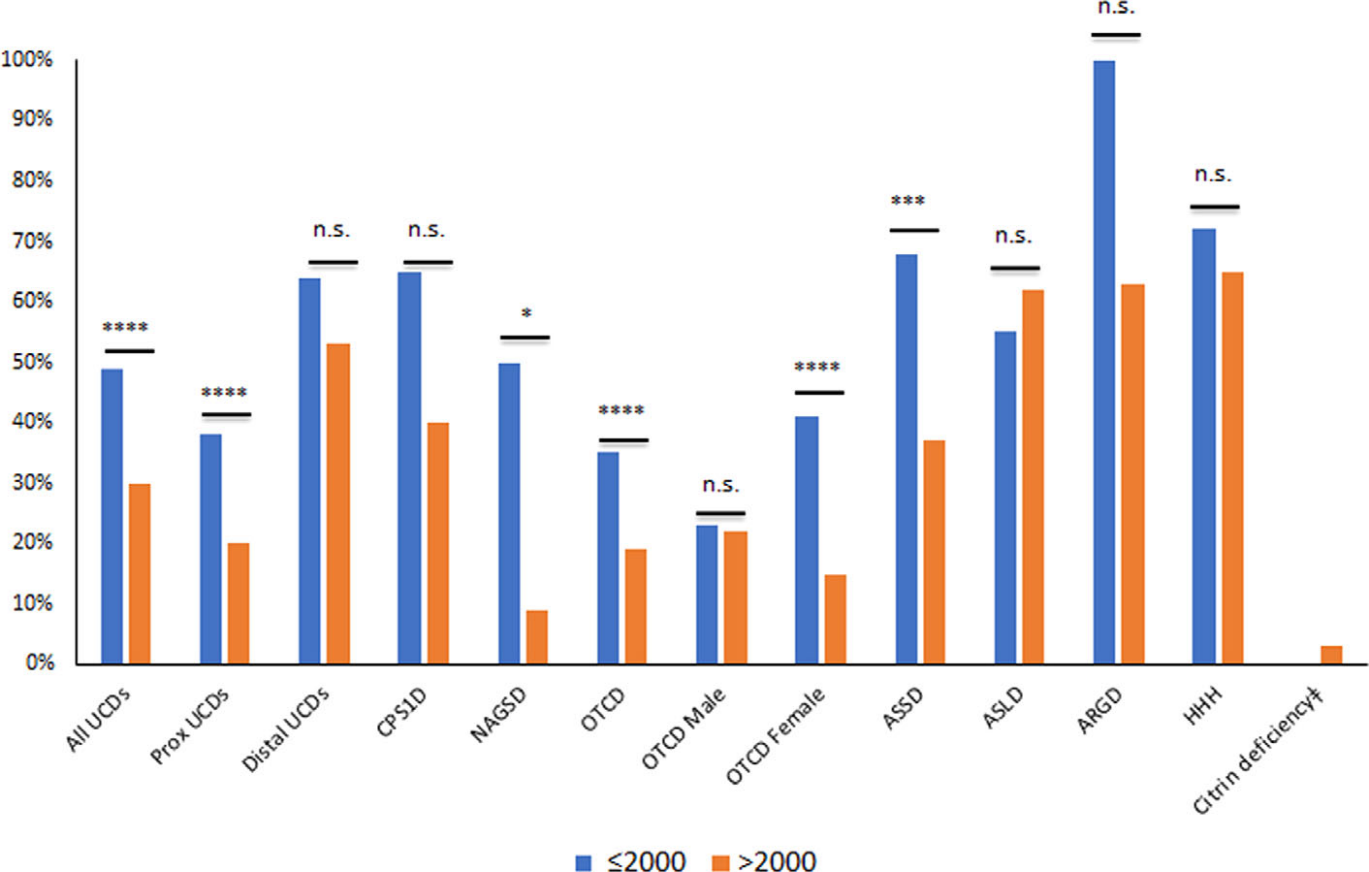
The rates of intellectual disabilities ≤2000 and >2000 for individual UCDs, proximal and distal UCDs and overall UCDs. *P* values for odds ratios for intellectual deficiencies >2000 compared to ≤2000 were calculated with the Fisher's exact test. *P < .05; **P < .01; ***P < .001; ****P < .0001; n.s., not significant. ǂData are only available after the year 2000 for Citrin deficiency. Abbreviations: ARGD, arginase deficiency; ASLD, argininosuccinate lyase deficiency; ASSD, argininosuccinate synthetase deficiency; CPS1D, carbamoyl phosphate synthetase 1 deficiency; HHH, hyperornithinemia‐hyperammonemia‐homocitrullinuria; NAGSD, *N*‐acetylglutamate synthase deficiency; OTCD, ornithine transcarbamylase deficiency

This review presents the neuropsychological manifestations in the eight urea cycle disorders. A “side‐by‐side” examination of published data over the years allows for an assessment of the degree to which changes in management have been successful in preventing intellectual disabilities or improving functioning. The pooled sample included data on cognitive abilities of 1649 individuals reported in 58 citations. A total of 556 patients (34%) functioned in the range of intellectual disabilities. The proportion of cases with intellectual disabilities declined in six disorders, ranging from 7% to 41% (as noted in Table 1). Overall, distal UCDs had poorer neurocognitive outcome than proximal UCDs (odds ratio = 3.82; CI = 3.005, 4.876; *P* < 0.001) (See Figure 2).

The most prominent improvement in neuropsychological outcome after year 2000 was observed in NAGSD. In NAGSD, the prevalence of intellectual disability declined from 50% to 9%, reflecting the efficacy of NCG therapy. The decline observed in females with OTCD (from 41% to 15%) is mainly due to nearly fourfold increase in total number of individuals, with the inclusion of asymptomatic heterozygotes. A similar decrease was not observed for males with OTCD (23% ≤2000 vs 22% after 2000). The improvement observed in cognitive outcomes of neonatal onsets CPS1D, ASSD, and ARGD may be related to effective management of severe HA. Despite similar treatment modalities, the prevalence of intellectual disabilities has increased among individuals with ASLD, which may be related to increased survival rates. The poor cognitive outcome of distal UCDs implicates an insidious impact of cumulative exposure to ammonia, arginine, and citrulline in the long term.^65^

Improved treatments have contributed to the survival of many infants, particularly males with OTCD who, nonetheless, experienced early irreversible neurological damage. Across studies, children with neonatal onset of symptoms, early liver failure, and higher levels of ammonia were more likely to have intellectual disabilities.^52^, ^53^, ^75^, ^106^ Early studies reported associations between cognitive outcomes and duration/severity of HA episodes and higher peak ammonia concentration.^45^, ^47^, ^48^, ^51^, ^107^ However, his review suggests that this generalization does not hold true in many cases (eg, Ref. 19).

While genotype was sometimes associated with neonatal vs later onset of symptoms, it did not reliably predict occurrence of intellectual disabilities in OTCD,^108^ CPS1,^16^, ^28^ HHH syndrome,^109^ citrullinemia type I,^66^ and probably in all the other UCDs, as well. Earlier treatment initiation (diet in less severe cases and ammonia scavengers or liver transplantation in severe cases) led to better outcomes in most studies,^7^, ^8^, ^45^, ^47^, ^61^, ^84^, ^110^ whereas lack of treatment or noncompliance with treatment resulted in poorer cognitive outcomes.^56^, ^67^, ^68^

Recent studies provide evidence for other variables that explain diversity of outcomes, including number of HA episodes, cumulative exposure to moderately abnormal biochemical levels,^65^ and other epigenetic factors or environmental stressors, such as alcohol consumption,^111^ menses,^25^ or pregnancy in women.^26^

The varied outcomes present challenges in answering the question of whether certain brain regions are more likely to be affected or whether the specific deficits noted reflect global intellectual and developmental impairment. A recent publication by Buerger et al^112^ argues for a global impact in OTCD.

Neuropsychological deficits in patients with UCD may be reversible with effective therapy, as was demonstrated in two 12‐month safety extension trials showing that glycerol phenylbutyrate improved executive function skills (including behavioral regulation, goal setting, planning, and self‐monitoring) when used as maintenance therapy in children.^110^ Liver transplantation was also associated with superior neurodevelopmental outcomes among patients with ammonia concentrations ≥300 μmol/L.^8^

The major limitations of this review are that most studies included heterogeneous samples and lacked control individuals for comparison. Studies including individuals identified by NBS were likely to detect far more cases of mild UCDs and may have led to misclassification of cases as late onset when treatment prevented early‐onset of symptoms. Almost none of the studies accounted for infants who died when diagnoses occurred before developmental assessment or post mortem. The pooled analyses provide only a rough picture of intellectual outcomes in UCDs, especially since not all cases were accounted for; the year 2000 as a dividing point for assumed treatment availability is arbitrary, and it is possible that the most affected or least affected individuals did not receive neuropsychological evaluations. Furthermore, patient samples may have included some overlap and some studies and case reports may have been missed.

Nonetheless, this review identified important design elements for natural history studies and eventual clinical trials. As shown by Rüegger et al,^19^ timing of diagnosis should be considered. In the group of patients whose diagnosis was delayed >1 year after symptom onset, 75% were affected by cognitive impairment compared with 46% if the delay was ≤1 year. Psychiatric and behavioral outcomes need to be assessed, as evidenced by reports noting psychosis, depression, anxiety, and/or social difficulties in every UCD. These symptoms may be due to neurological perturbations or to the experience of having a chronic illness. Attention‐deficit/hyperactivity disorder and behaviors associated with autism spectrum disorder were frequently reported, suggesting that these symptoms should also be assessed. Laboratory studies need to be included at regular intervals, even when the individual is not showing symptoms, so that correlations between severity of outcome and metabolic status can be reliably identified.^65^ Adequate functioning at school or within the community does not mean that the individual is unaffected by the UCD, as was apparent in studies of adults with OTCD,^55^, ^56^ thus reinforcing the need for regular neuropsychological evaluation.

Finally, this review supports efforts to develop novel therapies by highlighting the continued risks to neuropsychological functioning in individuals with UCDs. Except for citrin deficiencies, rates of intellectual disabilities remain high. Clearly, it is time to “raise the bar” in expectations for treatment. Earlier and more accurate diagnoses are now possible through genetic testing. NBS is becoming routine for many UCDs and is on the horizon for others. Perhaps most importantly, treatments that were once only imagined, such as enzyme replacement, genetic therapies, and drugs to enhance alternative metabolic pathways, are now possible.

## CONFLICT OF INTERESTS

S.E. Waisbren is a consultant to Horizon, Ultragenyx and Shire on the psychological attributes of urea cycle disorders. She received no financial support for work on this manuscript. She is also a member of the Urea Cycle Disorders Consortium (UCDC; U54HD061221), which is a part of the National Institutes of Health (NIH) Rare Disease Clinical Research Network (RDCRN), supported through collaboration between the Office of Rare Diseases Research (ORDR), the National Center for Advancing Translational Science (NCATS) and the Eunice Kennedy Shriver National Institute of Child Health and Human Development (NICHD). A.K. Stefanatos has nothing to disclose. T.M.Y. Kok is an employee of and has stock in Horizon. B. Ozturk‐Hismi received grant support from The Türkiye Bilimsel ve Teknolojik Araştirma Kurumu (TUBITAK‐1059B191601447).

## AUTHOR CONTRIBUTIONS

S.E. Waisbren, PhD, contributed to the design and literature review and was the primary author of the manuscript. A.K. Stefanatos, PhD, contributed to the literature review and helped edit the final manuscript. T.M.Y. Kok, BSc Pharm, contributed to the literature review and edited the manuscript. B. Ozturk‐Hismi, MD, contributed to the literature review, co‐authored sections of the manuscript, and edited the final manuscript.

## ETHICS APPROVAL/CONSENT

This article is a review of published literature and does not contain any new studies with human or animal subjects performed by the authors.

## GUARANTOR

S.E. Waisbren, PhD, is the guarantor for the article, accepts full responsibility for the work, and controlled the decision to publish.

## Supporting information

Data S1 Supporting Information
