# Supplementary material for: Neuropsychological attributes of urea cycle disorders: A systematic review of the literature
Source: J Inherit Metab Dis. 2019 Aug 1;42(6):1176–91. doi: 10.1002/jimd.12146 (PMC7250134; doi:10.1002/jimd.12146)
Supplement: Supplementary file 1 — Data S1 Supporting Information [file JIMD-42-1176-s001.docx]

**SUPPLEMENTARY MATERIAL**

**Table 1a: CPS1D**

| **First author,**  **Publication date** | **Country of origin** | **N _series_** | **N _CF_** | **N _ID_** | **% _ID_** | **N _neonatal_** | **N _late_** | **N _neonatal ID_** | **N _late ID_** | **Notes** |
| --- | --- | --- | --- | --- | --- | --- | --- | --- | --- | --- |
| **CPS1D (prior to and including 2000)** | | | | | | | | | | |
| Msall, 1984 | USA | 3 | 3 | 3 | 100% | 3 | 0 | 3 | 0 | IQ measured at age 19 ± 5 months;  1 died in follow-up |
| Msall, 1988 | USA | 1 | 1 | 1 | 100% | 0 | 1 | 0 | 1 | 21-year old patient treated for 9 years;  IQ reported as 10 |
| Maestri, 1991 | USA | 6 | 3 | 0 | 0% | 3 | 0 | 0 | 0 | Asymptomatic newborns, identified prenatally, siblings of affected individuals |
| Uchino, 1998 | Japan | 26 | 3 | 1 | 33% | 2 | 1 | 1 | 0 | Only 3 survived ≥5 years |
| Nassogne, 2005^*^  (1972-2000) | France | 14 | 6 | 5 | 83% | 5 | 1 | 4 | 1 | 6 children survived |
| Kosho, 2000 | Japan | 1 | 1 | 1 | 100% | 1 | 0 | 1 | 0 | Severe neonatal presentation; received hemodialysis and survived, but at 135 days, functioned at 3-month old level |
| **SUMMARY** | | **51** | **17** | **11** | **65%** | **14** | **3** | **9 (64%)** | **2 (67%)** |  |
| **CPS1D (after 2000)** | | | | | | | | | | |
| Wakutani, 2001 | Japan | 1 | 1 | 1 | 100% | 0 | 1 | 0 | 1 | Diagnosed at age 18, but earlier symptoms at age 13 |
| Eather, 2006 | Australia | 1 | 1 | 0 | 0% | 0 | 1 | 0 | 0 | Diagnosed during pregnancy at age 41 |
| Kurokawa, 2007 | Japan | 18 | 6 | 3 | 50% | 4 | 2 | 2 | 1 | 4 survived; 1 had liver transplantation |
| Klaus, 2009 | Germany  (Lebanese) | 2 | 1 | 0 | 0% | 0 | 1 | 0 | 0 | Late onset case  (Other case with neonatal-onset died) |
| Ono, 2009 | Japan | 1 | 1 | 0 | 0% | 1 | 0 | 0 | 0 | HA episode at 1 month; normal at 15 months |
| Serrano, 2010 | Spain | 2 | 2 | 1 | 50% | 0 | 2 | 0 | 1 | Late-onset children  (1 without ID had autistic behaviors that improved with treatment) |
| Bates, 2011 | Australia | 1 | 1 | 0 | 0% | 0 | 1 | 0 | 0 | Late-onset case, liver transplanted |
| Funghini, 2012 | Italy | 15 | 5 | 2 | 40% | 2 | 3 | 0 | 2 | 5 survived; One late-onset case without ID had liver transplantation |
| Martín-Hernández, 2014 | Spain | 2 | 2 | 2 | 100% | 1 | 1 | 1 | 1 | One died at age 9 in follow-up |
| Rüegger, 2014 | Austria, Germany, Netherlands, Switzerland, UK | 2 | 2 | 2 | 100% | 0 | 2 | 0 | 2 | Late onset cases |
| Foschi, 2015 | Italy | 1 | 1 | 0 | 0% | 0 | 1 | 0 | 0 | Late onset (age 3);  liver transplantation at age 17. |
| Ali, 2016 | Malaysia | 6 | 5 | 5 | 100% | 5 | 0 | 5 | 0 | Neonatal onset; 1 died as infant |
| Waisbren, 2016^**^ | USA, Canada, Germany, Switzerland | 24 | 24 | 5 | 21% | 20 | 3 | 4 | 2 | Infants (n=11) ID: 27%  Preschool children (n=5) ID: 0%  School age children (n=4) ID: 0%  Adults (n=4) ID: 50% |
| **SUMMARY** | | **76** | **52** | **21** | **40%** | **33** | **18** | **12 (36%)** | **10 (56%)** |  |
| **ODDS RATIO*** (95% CI, p-value)** | | | | **0.38 (0.098 – 1.311, p = 0.10)** | |  |  | **0.33 (0.068 – 1.383, p=0.11)** | **0.64 (0.009 – 14.443, p=1)** |  |

% _ID_, percentage of cases with intellectual disabilities; CPS1D, carbamoyl phosphate synthetase 1 deficiency; HA, hyperammonemia; ID, intellectual disability; IQ, intelligence quotient; N _CF_, number of cases reporting on cognitive functioning; N _ID_, number of cases reporting with intellectual disabilities; N _late_, number of late-onset cases reporting cognitive functioning; N _late ID_, number of late-onset cases reporting intellectual disabilities; N _neonatal_, number of neonatal-onset cases reporting cognitive functioning; N _neonatal ID_, number of neonatal-onset cases reporting intellectual disabilities; N _series_, number of cases in series; CI, confidence interval.

*Review of cases identified from 1972 to 2000

**Numbers reflect the neuropsychological evaluations performed in this longitudinal study.

***The estimated odds ratios (comparing intellectual deficiencies post-2000 to pre-2000), their 95% confidence intervals, and the p-values were obtained from the Fisher‘s exact test.

**Table 1b: NAGSD**

| **First author,**  **Publication year** | **Country of origin** | **N _series_** | **N _CF_** | **N _ID_** | **% _ID_** | **Notes** |
| --- | --- | --- | --- | --- | --- | --- |
| **NAGSD (prior to and including 2000)** | | | | | | |
| Schubiger, 1991 | Switzerland | 1 | 1 | 1 | 100% | Neonatal onset; treated with NCG since postnatal day 10; delayed development since age 6 months; died at age 9 |
| Pandya, 1991 | USA | 2 | 2 | 1 | 50% | Neonatal onset; treated with NCG; 2 brothers, one had normal development at 18 months and the other had developmental delay at 6 months |
| Burlina, 1992 | Italy | 1 | 1 | 1 | 100% | Late onset (2 months); treated without NCG; profound ID, cortical blindness, and movement disorder at age 2 |
| Vockley, 1992 | USA  (Taiwanese) | 1 | 1 | 0 | 0% | Late onset; treated without NCG; normal development at age 5 years, 7 months |
| Guffon, 1995 | France | 1 | 1 | 0 | 0% | Neonatal onset; treated with NCG; normal development at age 1 |
| Hinnie, 1997 | UK | 1 | 1 | 1 | 100% | Late onset; first symptoms at age 1.5 years, diagnosed at age 20; treated with NCG |
| Morris, 1998 | UK | 1 | 1 | 0 | 0% | Neonatal onset; treated with NCG; normal development at 20 months |
| Plecko,1998 | Austria | 1 | 1 | 0 | 0% | Late onset; recurrent episodes of psychosis and aggression in puberty; treated with NCG; IQ was 78 at 11 years |
| Forget, 1999 | Netherlands | 1 | 1 | 1 | 100% | Late onset; first symptom at age 2.7 years, diagnosed at age 4; treated with NCG |
| **SUMMARY** | | **10** | **10** | **5** | **50%** |  |
| **NAGSD (after 2000; all treated with NCG)** | | | | | | |
| Caldovic, 2005 | USA | 3 | 3 | 1 | 33% | Late onset; 2 sisters with normal cognition and a 33-year-old male who died during first coma with HA who needed special education in childhood |
| Nordenstrom, 2007 | Sweden | 1 | 1 | 0 | 0% | Neonatal onset; normal development at 3 years despite severe neonatal HA |
| Gessler, 2010 | Germany (Turkish) | 1 | 1 | 0 | 0% | Neonatal onset; normal development at age 4 years; neonatal HA but treated early |
| Martín-Hernández, 2014 | Spain | 1 | 1 | 0 | 0 | Late onset; presented with psychosis at age 12 |
| Rüegger, 2014 | Austria, Germany, Netherlands, Switzerland, UK | 1 | 1 | 0 | 0% | Late onset; diagnosed at age 13; normal development at age 27 |
| Sancho-Vaello* 2016 | Europe | 25 | 10 | 1 | 10% | Neonatal-onset (n=17); 1 had psychomotor delay, 3 had normal cognition (1 without NCG), and 6 died (1 after stopping NCG);  Late onset (n=8); 6 had normal cognition (4 without NCG) and 1 died. |
| Waisbren, 2016 | USA, Canada, Germany, Switzerland | 4 | 4 | 0 | 0% | 3 children, 1 adult; all had weaknesses in auditory memory; one adult had serious psychiatric and cognitive problems before diagnosis |
| Reigstad, 2017 | Norway | 1 | 1 | 0 | 0% | Neonatal onset; normal development at 7 weeks despite severe neonatal HA |
| **SUMMARY** | | **37** | **22** | **2** | **9%** |  |
| **ODDS RATIO** (95% CI, p-value)** | | | **0.11 (0.008 – 0.903, p=0.02)** | | | |

% _ID_, percentage of cases with intellectual disabilities; HA, hyperammonemia; IQ, intelligence quotient; NAGSD, *N*-acetylglutamate synthase deficiency; NCG, N-carbamylglutamate; N _CF_, number of cases reporting on cognitive functioning; NCG, *N*-carbamylglutamate; N _ID_, number of cases reporting with intellectual disabilities; N _series_, number of cases in series; CI, confidence interval.

*Only newly described cases in this study were included.

**The estimated odds ratios (comparing intellectual deficiencies post-2000 to pre-2000), their 95% confidence intervals, and the p-values were obtained from the Fisher‘s exact test.

**Table 1c: OTCD**

| **First author,**  **Publication year** | **Country of origin** | **N _series_** | | **N _CF_** | **N _ID_** | **% _ID_** | Notes | |
| --- | --- | --- | --- | --- | --- | --- | --- | --- |
| **OTCD (prior to and including 2000)** | | | | | | | | |
| Msall, 1984 | USA | 7 | | 5 | 2 | 40% | Males, symptomatic, untreated  (mean age 21 ± 5 months while undergoing IQ testing; 3 died) | |
| Rowe, 1986 | USA | 13 | | 13 | 5 | 38% | Females, symptomatic, mostly untreated  (few were on treatment while testing) | |
| Msall, 1988 | USA | 10 | | 10 | 5 | 50% | Females, symptomatic, treated  (2-year follow-up; age 7 ± 3 years; IQ of 61 ± 66) | |
|  |  | 21 | | 21 | 0 | 0% | Females, asymptomatic, untreated  (All adults; mean IQ of 108 ± 13; Verbal-performance IQ difference (VIQ>PIQ) is higher than healthy controls | |
| Maestri, 1991 | USA | 5 | | 4 | 0 | 0% | Males, identified by family history, treated prospectively  2 died in follow-up; 1 had liver transplantation and survived | |
| Maestri, 1996 | USA | 32 | | 23 | 10 | 43% | Females, symptomatic, treated  (5-year follow-up; 3 died; IQ levels declined in 4, despite treatment) | |
| Uchino, 1998 | Japan | 67 | | 28 | 3 | 11% | Males, treated  44 neonatal onset, 46 late onset, 2 asymptomatic; 28 survived ≥5 years | |
|  |  | 63 | | 19 | 5 | 26% | Females, treated  1 neonatal onset and 47 late onset; 19 survived ≥5 years | |
| Nassogne, 2005^*^  (1972-2000) | France | 112 | | 27 | 10 | 37% | Males (66 neonatal onset, untreated, 1 survived with ID; 46 late onset, 26 survived, 9 with ID) | |
|  |  | 38 | | 29 | 22 | 76% | Females (2 neonatal onset, untreated, both died; 36 late onset, 29 survived, 22 with ID) | |
| **Summary of Males** | | | **191** | **64** | **15** | **23%** |  | |
| **Summary of Females** | | | **177** | **115** | **47** | **41%** |  | |
| **Summary of All cases** | | | **368** | **179** | **62** | **35%** |  | |
| OTCD (after 2000; all treated) | | | | | | | | |
| Nicolaides, 2002 | UK | | 23 | 23 | 13 | 57% | Females (20 symptomatic, 13 with ID) | |
|  |  |  | 5 | 5 | 1 | 20% | Males (4 symptomatic, 1 with ID) | |
| Gyato, 2004 | USA | | 19 | 19 | 0 | 0% | Females. Asymptomatic (n=10) individuals had significantly higher IQ levels than symptomatic (n=9) ones. | |
| Keskinen, 2008 | Finland | | 20 | 14 | 1 | 7% | Males (4 neonatal onset, 16 late onset; 6 died) | |
|  |  |  | 10 | 9 | 4 | 44% | Females (1 died, 1 had liver transplantation) | |
| Serrano, 2010 | Spain | | 2 | 2 | 1 | 50% | Females, late onset | |
|  |  |  | 1 | 1 | 0 | 0% | Male, 8 year old, presented with absence seizures, hyperactivity, impulsiveness, anxiety | |
| Martín-Hernández, 2014 | Spain | | 26 | 24 | 7 | 29% | Males (7 neonatal onset, 15 late onset, 4 asymptomatic); 2 died |  |
|  |  |  | 41 | 41 | 22 | 54% | Females (2 neonatal onset, 37 late onset, 2 asymptomatic) |  |
| Rüegger, 2014 | Austria, Germany, Netherlands, Switzerland, UK | | 85 | 85 | 37 | 44% | Symptomatic (28 males and 57 females) | |
|  |  |  | 33 | 33 | 0 | 0% | Asymptomatic (10 males and 23 females) | |
| Waisbren, 2016^**^ | USA, Canada, Germany, Switzerland | | 25 | 25 | 3 | 12% | Infant girls | |
|  |  |  | 27 | 27 | 14 | 52% | Infant boys | |
|  |  |  | 31 | 31 | 0 | 0% | Preschool girls | |
|  |  |  | 15 | 15 | 2 | 13% | Preschool boys | |
|  |  |  | 97 | 97 | 8 | 8% | School-age girls | |
|  |  |  | 34 | 34 | 3 | 9% | School-age boys | |
|  |  |  | 156 | 156 | 8 | 5% | Adult women | |
|  |  |  | 25 | 25 | 4 | 16% | Adult men | |
| **Summary of Males** | | | **153** | **145** | **32** | **22%** | Excludes Rüegger cases since it was not specified how many males and females had ID | |
| **Summary of Females** | | | **404** | **403** | **59** | **15%** | Excludes Rüegger cases since it was not specified how many males and females had ID | |
| **Summary of All cases** | | | **675** | **666** | **128** | **19%** | Includes cases from Rüegger et al which was excluded from the numbers reported for males and females separately above. Because of this, the sum of males and females reported for all cases (>2000) is less than the number reported here for all cases. | |
| **MALES: ODDS RATIO*** (95% CI, p-value)** | | | |  | | | **0.93 (0.440 – 2.013, p=0.86)** | |
| **FEMALES: ODDS RATIO*** (95% CI, p-value)** | | | | | | | **0.25 (0.152 – 0.406, p<0.001)** | |
| **ALL CASES: ODDS RATIO*** (95% CI, p-value)** | | | | | | | **0.45 (0.308 – 0.659, p<0.001)** | |

% _ID_, percentage of cases with intellectual disabilities; ID, intellectual dysfunction; IQ, intelligence quotient; N _CF_, number of cases reporting on cognitive functioning; N _ID_, number of cases reporting with intellectual disabilities; N _series_, number of cases in series; OTCD, ornithine transcarbamylase deficiency; CI, confidence interval.

This table does not include previous reports emanating from the Urea Cycle Disorders Consortium since the cases were included in Waisbren 2016 (Ah Mew et al 2013; Batshaw et al 2014; Gropman et al 2008; Seminara et al 2010; Sprouse et al 2014).

*Review of cases identified from 1972 to 2000

**Numbers reflect the neuropsychological evaluations performed in this longitudinal study

*** The estimated odds ratios (comparing intellectual deficiencies post-2000 to pre-2000), their 95% confidence intervals, and the p-values were obtained from the Fisher‘s exact test.

**Table 1d: ASSD**

| **First author, Publication year** | **Country of origin** | | **N _series_** | | **N _CF_** | | **N _ID_** | | **% _ID_** | **Notes** |
| --- | --- | --- | --- | --- | --- | --- | --- | --- | --- | --- |
| ASSD/Citrullinemia type I (prior to and including 2000) | | | | | | | | | | |
| Msall, 1984 | USA | | 8 | | 8 | | 7 | | 88 | IQ measured at age 30 ± 4 months |
| Msall, 1988 | USA | | 1 | | 1 | | 1 | | 100 | Partial deficiency; tested at age 7 years |
| Uchino, 1998 | Japan | | 19 | | 9 | | 7 | | 78 | 14 neonatal onset and 5 late onset; 10 survived ≥5 years |
| Maestri, 1991 | USA | | 3 | | 3 | | 1 | | 33% | Asymptomatic patients diagnosed by NBS |
| Maestri, 1995 | USA | | 24 | | 15 | | 15 | | 100% | All neonatal onset; 9 died |
| Nassogne, 2005^*^  (1972-2000) | France | | 33 | | 14 | | 3 | | 21% | 26 neonatal onset (7 survived, 1 with ID)  7 late onset (7 survived, 2 with ID) |
| SUMMARY | | **88** | | **50** | | **34** | | **68%** | |  |
| ASSD/Citrullinemia type I (after 2000) | | | | | | | | | | |
| Serrano, 2010 | Spain | | 2 | | 2 | | 2 | | 100% | Late onset patients; 1 died (27 year old female, presented at postpartum day 2 with abnormal behaviors and altered mental status) |
| DeBie, 2011 | Canada | | 1 | | 1 | | 0 | | 0% | Neonatal onset; normal development at 8 years |
| Glamuzina, 2011 | New Zealand | | 3 | | 3 | | 0 | | 0% | Identified by NBS; not treated (mild form); followed until 22 months |
| Brunetti-Pierri, 2012 | USA | | 1 | | 1 | | 1 | | 100% | 31-year-old female with neonatal onset |
| Lee, 2013 | Republic of Korea | | 20 | | 14 | | 9 | | 64% | 14 neonatal onset (9 survived, 8 with ID); 3 late onset (2 survived, 1 with ID); 3 asymptomatic (none had ID) |
| Martín-Hernández, 2014 | Spain | | 22 | | 21 | | 14 | | 67% | 14 neonatal onset (ID: 100%) , 4 late onset (ID: 25%) , 4 asymptomatic (ID: 0%) |
| Rüegger, 2014 | Austria, Germany, Netherlands, Switzerland, UK | | 43 | | 43 | | 7 | | 16% | 28 asymptomatic (ID: 0%); 15 symptomatic (ID: 47%); 2 of 7 with ID found by NBS |
| Waisbren, 2018 | USA, Canada, Germany, Switzerland | | 64 | | 52 | | 18 | | 35% | 17 neonatal onset, 47 late onset; 13 had liver transplantation; 13 identified by NBS |
| **SUMMARY** | | **156** | | **137** | | **51** | | **37%** | |  |
| **ODDS RATIO** (95% CI, p-value)** | | | | **0.28 (0.131 – 0.583, p<0.001)** | | | | | | |

% _ID_, percentage of cases with intellectual disabilities; ASSD, argininosuccinate synthetase deficiency; ID, intellectual dysfunction; IQ, intelligence quotient; NBS, newborn screening; N _CF_, number of cases reporting on cognitive functioning; N _ID_, number of cases reporting with intellectual disabilities; N _series_, number of cases in series.

*Review of cases identified from 1972 to 2000

**The estimated odds ratios (comparing intellectual deficiencies post-2000 to pre-2000), their 95% confidence intervals, and the p-values were obtained from the Fisher‘s exact test.

**Table 1e: ASLD/ASA**

| **First author,**  **Publication year** | **Country of origin** | | **N _series_** | **N _CF_** | **N _ID_** | **% _ID_** | **Notes** |
| --- | --- | --- | --- | --- | --- | --- | --- |
| ASLD/ASA (prior to and including 2000) | | | | | | | |
| Msall, 1984 | USA | | 8 | 8 | 7 | 88% | Untreated; mean age 41 ± 7 months at IQ testing |
| Msall, 1988 | USA | | 1 | 1 | 1 | 100% | Partial deficiency |
| Uchino, 1998 | Japan | | 12 | 6 | 5 | 83% | 8 neonatal onset, 2 late onset, and 2 prospectively diagnosed; 6 survived ≥ 5 years |
| Maestri, 1991 | USA | | 1 | 1 | 1 | 100% | Identified and treated prospectively |
| Widhalm, 1992 | Austria | | 22 | 12 | 0 | 0% | Treated asymptomatic patients detected with NBS; DQ/IQ of 92–132 (22 patients, ages 1–9 years) |
| Gerrits, 1993 | Netherlands | | 3 | 2 | 1 | 50% | Late-onset untreated patients; one had mild ID and one had IQ of 71 at age 5 |
| Nassogne, 2005^*^ (1972-2000) | France | | 20 | 12 | 8 | 67% | 14 neonatal onset (8 died, ID: 83%) , 6 late onset (ID: 50%) |
| **SUMMARY** | | | **67** | **42** | **23** | **55%** |  |
| ASLD/ ASA (after 2000) | | | | | | | |
| Keskinen, 2008 | Finland | | 20 | 15 | 9 | 60% | 8 died by the time of the study (ages 1–68 years). Of the 15 whose development was recorded, 9 had moderate to severe ID |
| Ficicioglu, 2009 | USA | | 13 | 13 | 0 | 0% | Asymptomatic patients detected on NBS |
| Mercimek-Mahmutoglu, 2010 | Austria | | 23 | 17 | 1 | 6% | Asymptomatic patients detected on NBS |
| Serrano, 2010 | Spain | | 2 | 2 | 2 | 100% | Late onset patients |
| Grioni, 2011 | Italy | | 11 | 11 | 9 | 82% | Among 9 with ID, 6 had seizures |
| Martín-Hernández, 2014 | | Spain | 10 | 10 | 8 | 80% | 2 neonatal onset (ID: 100%), 6 late onset (ID: 100%) , 2 asymptomatic (ID: 0%) |
| Rüegger, 2014 | Austria, Germany, Netherlands, Switzerland, UK | | 31 | 31 | 20 | 65% | 11 asymptomatic (ID: 0%); 20 symptomatic (ID: 100%) |
| Ganetzky, 2017 | USA | | 1 | 1 | 1 | 100% | Symptomatic case missed by NBS |
| Baruteau, 2017 | UK | | 56 | 52 | 48 | 92% | 23 neonatal onset, 23 late onset, and 10 perinatally diagnosed |
| Waisbren, 2018 | USA, Canada, Germany, Switzerland | | 65 | 60 | 33 | 55% | 15 neonatal onset, 50 late onset; 11 had liver transplantation; 19 identified through NBS |
| SUMMARY | | | **232** | **212** | **131** | **62%** |  |
| ODDS RATIO**(95% CI, p-value) | | | | **1.33 (0.644 – 2.743, p=0.39)** | | |  |

% _ID_, percentage of cases with intellectual disabilities; ASLD, argininosuccinate lyase deficiency; DQ, developmental quotient; ID, intellectual dysfunction; IQ, intelligence quotient; NBS, newborn screening; N _CF_, number of cases reporting on cognitive functioning; N _ID_, number of cases reporting with intellectual disabilities; N _series_, number of cases in series.

*Review of cases identified from 1972 to 2000

** The estimated odds ratios (comparing intellectual deficiencies post-2000 to pre-2000), their 95% confidence intervals, and the p-values were obtained from the Fisher‘s exact test

**Table 1f: ARGD**

| **First author, publication year** | **Country of origin** | **N _series_** | **N _CF_** | **N _ID_** | **% _ID_** | **Notes** |
| --- | --- | --- | --- | --- | --- | --- |
| ARGD (prior to and including 2000) | | | | | | |
| Msall, 1988 | USA | 1 | 1 | 1 | 100% | Partial deficiency |
| Uchino, 1998 | Japan | 7 | 3 | 3 | 100% | 3 survived |
| **SUMMARY** | | **8** | **4** | **4** | **100%** |  |
| ARGD (after 2000) | | | | | | |
| Görker, 2005 | Turkey | 1 | 1 | 0 | 0% | 4-year-old girl with autistic-like presentation; improved with treatment (authors also suggested OTCD, but clinical picture was ARGD) |
| Baranello, 2014 | Italy | 1 | 1 | 0 | 0% | 10-year-old boy with progressive spastic paraparesis |
| Rüegger, 2014 | Switzerland, Germany, Austria, UK, Netherlands | 8 | 8 | 7 | 88% | All symptomatic; 7 had seizures, 4 had spasticity |
| Martín-Hernández, 2014 | Spain | 2 | 2 | 0 | 0% | 1 neonatal onset and 1 diagnosed through NBS |
| Waisbren , 2018 | USA, Canada, Germany, Switzerland | 16 | 12 | 8 | 67% | All late onset |
| **SUMMARY** | | **28** | **24** | **15** | **63%** |  |
| **Odds Ratio^*^ (95% CI, p-value)** | | | **0 (0 to 3.153, p=0.3)** | | |  |

% _ID_, percentage of cases with intellectual disabilities; ARGD, arginase deficiency; NBS, newborn screening; N _CF_, number of cases reporting on cognitive functioning; N _ID_, number of cases reporting with intellectual disabilities; N _series_, number of cases in series; OTCD, ornithine transcarbamylase deficiency; CI, confidence interval.

* The estimated odds ratios (comparing intellectual deficiencies post-2000 to pre-2000), their 95% confidence intervals, and the p-values were obtained from the Fisher‘s exact test.

**Table 1g: Mitochondrial transporter deficiencies**

| **First author, publication year** | Country of origin | **N _series_** | **N _CF_** | **N _ID_** | **% _ID_** | **Notes** |
| --- | --- | --- | --- | --- | --- | --- |
| **Citrin deficiency (prior to and including 2000)** | | | | | | |
| No studies reporting cognitive outcomes were found for Citrin deficiency ≤ 2000. | | | | | | |
| **Citrin deficiency (after 2000)** | | | | | | |
| Ikeda, 2001 | Japan | 10 | 9 | 1 | 11% | All had CTLN2; 7 had liver transplantation |
| Ohura, 2007 | Japan | 75 | 75 | 2 | 3% | All had NICCD |
| Song, 2011 | China | 51 | 51 | 1 | 2% | 49 with NICCD and 2 with failure to thrive and dyslipidemia caused by citrin deficiency |
| Waisbren, 2016 | USA, Canada, Germany, Switzerland | 2 | 2 | 0 | 0% | Both with NICCD |
| Summary | | **138** | **137** | **4** | **3%** |  |
| HHH Syndrome (prior to and including 2000) | | | | | | |
| Martinelli, 2015^*^ | Global (literature review) | **38** | **32** | **23** | **72%** | 3 neonatal-onset cases, 13 late-onset cases (onset unreported in 22); 2 died |
| HHH Syndrome (after 2000) | | | | | | |
| Rüegger, 2014 | Switzerland, Germany, Austria, UK, Netherlands | 2 | 2 | 1 | 50% | 2 survived |
| Martinelli, 2015^*^ | Global (literature review) | 73 | 53 | 35 | 66% | 9 neonatal-onset cases, 29 late-onset cases (onset unreported in 35); 5 died. |
| Waisbren, 2016 | USA, Canada, Germany, Switzerland | 7 | 7 | 3 | 43% | 1 adult had a decline in IQ from 100 to 84 over 5 years |
| Guan, 2017 | China | 3 | 3 | 3 | 100% | 1 had liver transplantation |
| **SUMMARY** | | **85** | **65** | **42** | **65%** |  |
| **Odds Ratio^**^ (95% CI, p-value)** | | | **0.72 (0.249 – 1.947, p=0.50)** | | | |

% _ID_, percentage of cases with intellectual disabilities; CTLN2, citrullinemia type II; HHH, hyperornithinemia-hyperammonemia-homocitrullinuria; N _CF_, number of cases reporting on cognitive functioning; NICCD, neonatal intrahepatic cholestasis caused by citrin deficiency; N _ID_, number of cases reporting with intellectual disabilities; N _series_, number of cases in series.

*Literature review; cases are pooled according to publication year

** The estimated odds ratios (comparing intellectual deficiencies post-2000 to pre-2000), their 95% confidence intervals, and the p-values were obtained from the Fisher‘s exact test

REFERENCES

Ah Mew N, Krivitzky L, McCarter R, Batshaw M, Tuchman M (2013) Clinical outcomes of neonatal onset proximal versus distal urea cycle disorders do not differ. *J Pediatr* **162**:324–329.e321.

Batshaw ML, Tuchman M, Summar M, Seminara J. A longitudinal study of urea cycle disorders. *Mol Genet Metab*. 2014; **113**:127-130.

Burgoon JK, Berger CR, Waldron VR. Mindfulness and interpersonal communication. *J Social Issues*. 2000; **56**:105-127.

Caldovic L, Morizono H, Panglao MG, et al. Late onset N-acetylglutamate synthase deficiency caused by hypomorphic alleles. *Hum Mutat*. 2005; **25**:293-298.

Enns GM. Neurologic damage and neurocognitive dysfunction in urea cycle disorders. *Semin Pediatr Neurol*. 2008; **15**:132-139.

Gerrits GP, Gabreels FJ, Monnens LA, et al. Argininosuccinic aciduria: clinical and biochemical findings in three children with the late onset form, with special emphasis on cerebrospinal fluid findings of amino acids and pyrimidines. *Neuropediatrics*. 1993; **24**:15-18.

Gropman AL, Seltzer RR, Yudkoff M, Sawyer A, VanMeter J, Fricke ST. 1H MRS allows brain phenotype differentiation in sisters with late onset ornithine transcarbamylase deficiency (OTCD) and discordant clinical presentations. *Mol Genet Metab*. 2008; **94**:52-60.

Hediger N, Landolt MA, Diez-Fernandez C, Huemer M, Haberle J. The impact of ammonia levels and dialysis on outcome in 202 patients with neonatal onset urea cycle disorders. *J Inherit Metab Dis*. 2018; **41**:689-698.

Ikeda S, Yazaki M, Takei Y, et al. Type II (adult onset) citrullinaemia: clinical pictures and the therapeutic effect of liver transplantation. *J Neurol Neurosurg Psychiatry*. 2001; **71**:663-670.

Maestri NE, Brusilow SW, Clissold DB, Bassett SS. Long-term treatment of girls with ornithine transcarbamylase deficiency. *N Engl J Med*. 1996; **335**:855-859.

Maestri NE, Hauser ER, Bartholomew D, Brusilow SW. Prospective treatment of urea cycle disorders. *J Pediatr*. 1991; **119**:923-928.

Ohura T, Kobayashi K, Tazawa Y, et al. Clinical pictures of 75 patients with neonatal intrahepatic cholestasis caused by citrin deficiency (NICCD). *J Inherit Metab Dis*. 2007; **30**:139-144.

Posset R, Garbade SF, Boy N, et al. Transatlantic combined and comparative data analysis of 1095 patients with urea cycle disorders-asuccessful strategy for clinical research of rare diseases. *J Inherit Metab Dis*. 2018; **42**:93-106.

Saudubray JM, Touati G, Delonlay P, et al. Liver transplantation in urea cycle disorders. *Eur J Pediatr*. 1999; **158**(suppl 2):S55-S59.

Widhalm K, Koch S, Scheibenreiter S, et al. Long-term follow-up of 12 patients with the late-onset variant of argininosuccinic acid lyase deficiency: no impairment of intellectual and psychomotor development during therapy. *Pediatrics*.

1992; **89**:1182-1184.
